# The 2nd DBCLS BioHackathon: interoperable bioinformatics Web services for integrated applications

**DOI:** 10.1186/2041-1480-2-4

**Published:** 2011-08-02

**Authors:** Toshiaki Katayama, Mark D Wilkinson, Rutger Vos, Takeshi Kawashima, Shuichi Kawashima, Mitsuteru Nakao, Yasunori Yamamoto, Hong-Woo Chun, Atsuko Yamaguchi, Shin Kawano, Jan Aerts, Kiyoko F Aoki-Kinoshita, Kazuharu Arakawa, Bruno Aranda, Raoul JP Bonnal, José M Fernández, Takatomo Fujisawa, Paul MK Gordon, Naohisa Goto, Syed Haider, Todd Harris, Takashi Hatakeyama, Isaac Ho, Masumi Itoh, Arek Kasprzyk, Nobuhiro Kido, Young-Joo Kim, Akira R Kinjo, Fumikazu Konishi, Yulia Kovarskaya, Greg von Kuster, Alberto Labarga, Vachiranee Limviphuvadh, Luke McCarthy, Yasukazu Nakamura, Yunsun Nam, Kozo Nishida, Kunihiro Nishimura, Tatsuya Nishizawa, Soichi Ogishima, Tom Oinn, Shinobu Okamoto, Shujiro Okuda, Keiichiro Ono, Kazuki Oshita, Keun-Joon Park, Nicholas Putnam, Martin Senger, Jessica Severin, Yasumasa Shigemoto, Hideaki Sugawara, James Taylor, Oswaldo Trelles, Chisato Yamasaki, Riu Yamashita, Noriyuki Satoh, Toshihisa Takagi

**Affiliations:** 1 Database Center for Life Science, Research Organization of Information and Systems, 2-11-16 Yayoi, Bunkyo-ku, Tokyo, 113-0032, Japan

## Abstract

**Background:**

The interaction between biological researchers and the bioinformatics tools they use is still hampered by incomplete interoperability between such tools. To ensure interoperability initiatives are effectively deployed, end-user applications need to be aware of, and support, best practices and standards. Here, we report on an initiative in which software developers and genome biologists came together to explore and raise awareness of these issues: BioHackathon 2009.

**Results:**

Developers in attendance came from diverse backgrounds, with experts in Web services, workflow tools, text mining and visualization. Genome biologists provided expertise and exemplar data from the domains of sequence and pathway analysis and glyco-informatics. One goal of the meeting was to evaluate the ability to address real world use cases in these domains using the tools that the developers represented. This resulted in i) a workflow to annotate 100,000 sequences from an invertebrate species; ii) an integrated system for analysis of the transcription factor binding sites (TFBSs) enriched based on differential gene expression data obtained from a microarray experiment; iii) a workflow to enumerate putative physical protein interactions among enzymes in a metabolic pathway using protein structure data; iv) a workflow to analyze glyco-gene-related diseases by searching for human homologs of glyco-genes in other species, such as fruit flies, and retrieving their phenotype-annotated SNPs.

**Conclusions:**

Beyond deriving prototype solutions for each use-case, a second major purpose of the BioHackathon was to highlight areas of insufficiency. We discuss the issues raised by our exploration of the problem/solution space, concluding that there are still problems with the way Web services are modeled and annotated, including: i) the absence of several useful data or analysis functions in the Web service "space"; ii) the lack of documentation of methods; iii) lack of compliance with the SOAP/WSDL specification among and between various programming-language libraries; and iv) incompatibility between various bioinformatics data formats. Although it was still difficult to solve real world problems posed to the developers by the biological researchers in attendance because of these problems, we note the promise of addressing these issues within a semantic framework.

## Background

Life Sciences are facing a new era where unprecedented amounts of genomic-scale data are produced daily. To handle the data from, for example, next-generation DNA sequencers, each major genomics institute has been developing local data-management tools and analytical pipelines. Even small laboratories are now able to plan large sequencing projects due to significantly reduced sequencing costs and the availability of commodity or contract-based sequencing centers. However, once in the hands of biological researchers, these sequences must still undergo significant analyses to yield novel discoveries, and research groups frequently create their own in-house analytical pipelines. Such "boutique" analyses can, therefore, become a significant bottleneck for genomics research when data sets are large. To overcome this, it is necessary to improve the interaction between biological researchers and the bioinformatics tools they require. To address these needs, researchers at the Database Center for Life Science [1] have initiated a series of BioHackathons that bring together researchers from the global bioinformatics and genomics communities to address specific problems in a collaborative setting.

The first DBCLS BioHackathon [2] focused on standardization of bioinformatics Web services, and in particular on standardizing data-exchange formats to increase interoperability in support of simplified bioinformatics workflow construction. Nevertheless, to make these interoperability initiatives operational for Life Science researchers - genome biologists in particular - end-user applications need to be aware of, and support, these best practices and standards. To this end, the second BioHackathon gathered developers of mashup services and Web service providers together with genome biologists who provided exemplar data to evaluate the ability of participating services and interfaces to address real world use cases.

**Figure 1:**
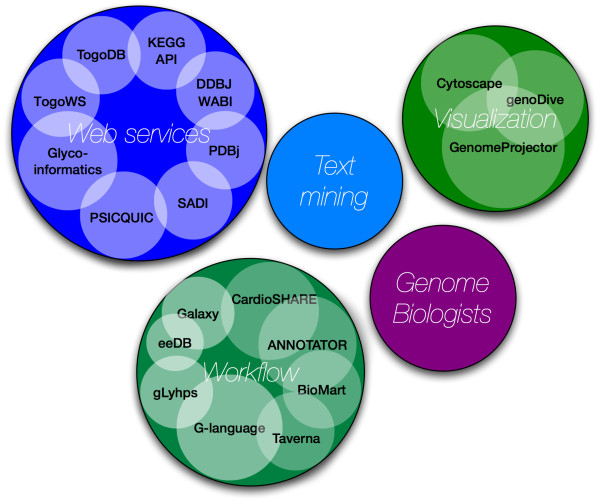
**Attendees of the DBCLS BioHackathon 2009**. The BioHackathon 2009 was attended by representatives from projects in Web services, Text Mining, Visualization and Workflow development, in addition to genome biologists who provided real-world use cases from their research.

The second DBCLS BioHackathon took place March 11-15, 2009 in Japan, jointly hosted by the DBCLS and the Okinawa Institute of Science and Technology (OIST [3]). DBCLS is a national project responsible for developing services to integrate bioinformatics resources, while OIST hosts a research unit focusing on marine genomics. The researchers and developers attending the second DBCLS BioHackathon represented key resources and projects within a number of related domains (Figure 1):

• **Web services **Among participating Web service projects were a number of key Japanese projects, including providers of database services (DDBJ WABI [4-6], PDBj [7,8], KEGG API [9-12]) and providers of data integration and generic APIs. Examples of the latter are the TogoDB [13] database services which are exposed through the TogoWS [14,15] architecture. There were also representatives of the G-language Genome Analysis Environment [16], which is a set of Perl libraries for genome sequence analysis that is compatible with BioPerl, and equipped with several software interfaces (interactive Perl/UNIX shell with persistent data, AJAX Web GUI, Perl API). In addition, there were representatives of projects developing domain-specific Web services and standards for data formats and exchange protocols (PSICQUIC [17], Glycoinformatics [18]). Lastly, there were representatives from the Semantic Automated Discovery and Integration framework (SADI [19,20]).

• **Workflows **- While individual data and analytical tools are made available through Web services, biological discovery generally requires executing a series of data retrieval, integration, and analysis steps. Thus, it is important to have environments within which Web services can be easily pipelined, and where high-throughput data can be processed without manual intervention. Among the projects that enable this were participants from ANNOTATOR [21], MOWserv [22]/jORCA [23], IWWE&M [24], Taverna [25] and RIKEN Life Science Accelerator (EdgeExpressDB [26]). In addition, there were representatives from BioMart [27], Galaxy [28] and SHARE [29]. BioMart is a query-oriented data management system that is particularly suited for providing 'data mining'-like searches of complex descriptive data. Galaxy is an interactive platform to obtain, process, and analyze biological data using a variety of server-side tools. The SHARE client is designed specifically for the SADI Web service framework, where it parses a SPARQL query and automatically maps query clauses to appropriate Web services, thus automatically creating a query-answering workflow.

• **Text mining **- Although a significant portion of our knowledge about life science is stored in a vast number of papers, few linkages exist between the rich knowledge "hidden" in the scientific literature and the rich data catalogued in our databases. To bridge them automatically, we first need to annotate those papers manually. Among the BioHackathon participants were researchers/developers from such annotation projects, namely Kazusa Annotation [30], and Allie [31].

• **Visualization **- Biological data visualization involves not only providing effective abstractions of vast amounts of data, but also effective and facile ways to find, retrieve and store data as-needed by the biologist for a fast and complete visual exploration. To achieve this, both data providers and tool developers need to work collaboratively as much of the data that we need to visualize is complex and dispersed among a wide variety of non-coordinating providers. To complicate the field even further, biologists work at a wide range of scales as they attempt to discover new insights - from meta-genomic, multi-genome comparisons, to single genomes, to interactome, to single gene or protein, to SNP information. Each scale and type of data requires a different approach to visualization. At the BioHackathon there were representatives from a number of visualization projects. The genoDive [32] is a genome browser for viewing schematic genome structures and associated information in 3D space with the ability to execute a "semantic zoom" (i.e. a zoom which is aware of its context). This novel representation of genomic information provides an alternative to the more common 2D-track displays. GenomeProjector [33] is a tool based on the Google Map API to combine different views for the genomic data in context of genome, plasmid, pathway and DNA walk. In addition, there were representatives from Cytoscape [34,35] and GBrowse [36,37]. GBrowse is a genome viewer and Cytoscape is a visualizer of the biomolecular interaction networks.

The participants from these different domains collaboratively challenged real world issues in genome biology based on use cases described in the Methods section. However, beyond deriving prototype solutions for each use case, another major purpose of the BioHackathon series is to identify problems and weaknesses in current technologies, such that "bio-hackers" can return to their respective groups with a clear focus on areas of immediate need. As such, we conclude the paper with an extensive discussion of the issues raised by our exploration of the problem/solution space; in particular, issues related to data formats, the complexity of Web service interoperability, and the need for semantics in bioinformatics data and tools.

## Methods

The BioHackathon followed a use-case-driven model. First, genome biologists having developmental, evolutionary, genetic and medical interests explained their data retrieval, integration and analysis requirements. From these, four use-cases were developed spanning three general domains of genomics data.

To address the use cases outlined in the Table 1, developers of the end-user client tools ANNOTATOR, Galaxy, BioMart, TogoDB, jORCA and Taverna presented the features of their projects at the BioHackathon and how they might be utilized to solve the use cases, and then collaboratively worked toward resolution for each. The following sections summarize successes and failures for each use case in the context of the tool or framework being applied, and an evaluation of each tool in comparison to alternatives. In general, the participants achieved operational results within the time span of the BioHackathon, which demonstrates some of the strengths of Web services in being accessible from any of the participants' computers and, in principle, programmable in any language.

**Table 1:** Summary of technical problems and solutions for each use case

Use Case 1	Annotation of 100,000 invertebrate ESTs
Task	A researcher needs to annotate 100,000 sequences obtained from an invertebrate species and also needs to provide the result as a public database.

Strategy	Annotate sequences by similarity and complement these annotations for sequences showing no similarity by integrated analysis tools. Then, store the results into BioMart or TogoDB to make the database publicly available.

Problem	Needed to identify which tool was most suitable for each step. Some tools turned out to require very long time for execution. The resulting annotations needed to be archived in a database and made accessible on the Web.

Solution	Firstly, use relatively fast tools like Blast2GO and KAAS then use ANNOTATOR for limted number of sequences. BioMart is suitable for integration of remote BioMart resources like Ensembl, while TogoDB can be used to host databases without installation. Both database systems are accessible through the Web service interface for workflow tools like jORCA and Taverna.

Tools	Blast2GO, KAAS, ANNOTATOR, BioMart, TogoDB, TogoWS, jORCA, Taverna

Databases	Ensembl, BioMart, KEGG

**Use Case 2**	**TFBS enrichment within differential microarray gene expression data**

Task	Identify SNPs in transcription factor binding sites and visualize the result as a genome browser.

Strategy	Retrieve SNP and TSS datasets through the DAS protocol, then compute enrichment and export results for a DAS viewer.

Problem	Needed to integrate information from multiple databases and needed to customize the visualization.

Solution	Developed a custom-made prediction system for the data obtained from DAS sources, then customize the Ajax DAS viewer to show the result in a genomic view.

Tools	BioDAS, Ajax DAS viewer

Databases	FESD II, DBTSS

**Use Case 3**	**Protein interactions among enzymes in a KEGG metabolic pathway**

Task	Predict interacting pairs of proteins in a given metabolic pathway.

Strategy	Retrieve enzymes from a specified pathway and search pairs of homologous proteins forming complexes in a strucuture database.

Problem	Found version incompatilibity of the server and client implementations of SOAP protocol. Non-standard BLAST output format was returned by PDBj Web service. There were no Web services to calculate phylogenetic profile.

Solution	Switch programming languages according to the service in use. Programs are written to parse BLAST results and to generate a phylogenetic profile.

Tools	Java, OCaml, Perl, Ruby, BLAST, DDBJ WABI, PDBj Mine, KEGG API

Databases	DDBJ, KEGG, PDBj, UniProt

**Use Case 4**	**Analyzing glyco-gene-related diseases**

Task	Find human diseases which are potentially related to SNPs and glycans.

Stragety	Retrieve disease genes and search for homologs in other organisms to which glyco-gene interactions are recoreded, then search for epitopes to identify glycans and retrieve their structures.

Problem	No Web service existed to query GlycoEpitopeDB and to convert a glycan structure in IUPAC format into KCF format. The output of OMIM search was in XML including entries which did not contain SNPs.

Solution	Implemented and registered BioMoby compliant Web services. Wrote custom BeanShell script for a Taverna workflow.

Tools	Taverna, BioMoby, KEGG API

Databases	OMIM, H-InvDB, GlycoEpitopeDB, RINGS, Consortium for Functional Glycomics, GlycomeDB, GlycoGene DataBase, KEGG

### Use Case 1 - Annotation of 100,000 invertebrate ESTs

**Figure 2:**
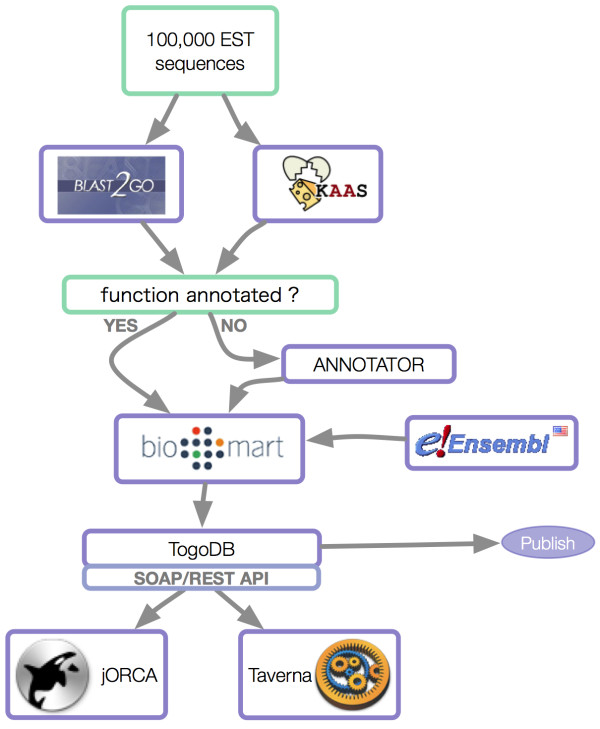
**Workflow to annotate large sets of ESTs**. Sequences are firstly annotated using high-throughput systems (e.g. Blast2GO, KAAS). Remaining difficult-to-annotate sequences are subsequently passed through ANNOTATOR for deeper analysis. The combined sequences are then joined with related annotations in the remote Ensembl database using BioMart and exposed through TogoDB such that they can be consumed by workflow managers (e.g. jORCA or Taverna) as TogoWS services.

As the number of sequences in this case is relatively large, sequences should first be annotated using a high-throughput system such as Blast2GO [38] or KAAS [39]. BLAST2GO is a tool to annotate many sequences at once with gene ontology (GO) definitions based on BLAST sequence similarity. KEGG Automatic Annotation Server (KAAS) is a service for functional annotation of sequences by assigning them to KEGG pathways. After using these tools, ANNOTATOR can be used to perform deeper analysis on the remaining difficult-to-annotate sequences, as ANNOTATOR provides highly detailed analysis. Specifically, ANNOTATOR provides functionality to predict protein function based on physicochemical characteristics such as secondary structure of a protein or prediction of transmembrane regions, which can give insight to predict molecular characteristics and cellular functions of the protein. To accomplish this, ANNOTATOR integrates various bioinformatics algorithms and Web services that sometimes take very long time to run. This is why the prototype used ANNOTATOR for only those proteins that are difficult to annotate by sequence similarity. BioMart can subsequently be used to join annotated sequences stored in a local BioMart server with related annotation in the remote Ensembl [40] database. Finally, finished annotations can be published as a simple database using TogoDB and the result can be integrated into workflow managers like jORCA or Taverna through (TogoWS) Web services. Figure 2 shows the steps of this workflow.

### Use Case 2 - TFBS enrichment within differential microarray gene expression data

**Figure 3:**
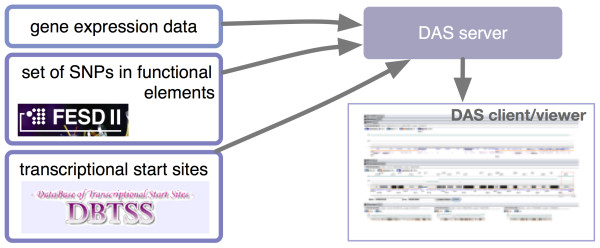
**System to enrich TFBSs with differential expression data**. Data on transcriptional start sites and on functional element SNPs are combined using distributed annotation system (DAS) protocol layers for the DBTSS and FESD II databases, respectively. Providing a list of genes or proteins (e.g. gene expression data), enrichment can then be computed and exposed using a DAS viewer.

A researcher needs to construct an integrated system for analysis of the transcription factor binding sites (TFBSs) enriched based on differential gene expression data obtained from a microarray experiment. The goal is to explore possible variations in the genomic sequence that could explain the differences in expression. To address this use case, participants identified the Functional Element SNP database (FESD II [41]), which is a Web-based system for selecting sets of SNPs in putative functional elements in human genes. It provides sets of SNPs located in 10 different functional elements: promoter regions, CpG islands, 5'UTRs (untranslated regions), translation start sites, splice sites, coding exons, introns, translation stop sites, poly adenylation signals (PASes), and 3'UTRs. It would then be necessary to integrate FESD data with data from the database of transcriptional start sites (DBTSS [42]) which is based on experimentally-determined 5'-end sequences of full-length cDNAs. To accomplish this, participants chose the distributed annotation system (DAS [43]) protocol to gather sequence annotations from distant servers and implemented a DAS layer for FESD II and DBTSS. As a tangible outcome, participants developed TFBS prediction systems that compute the enrichment of the TFBS for a list of genes or proteins, and present the results using an Ajax DAS viewer [44]. The components of this system are shown in Figure 3.

### Use Case 3 - Protein interactions among enzymes in a KEGG metabolic pathway

**Figure 4:**
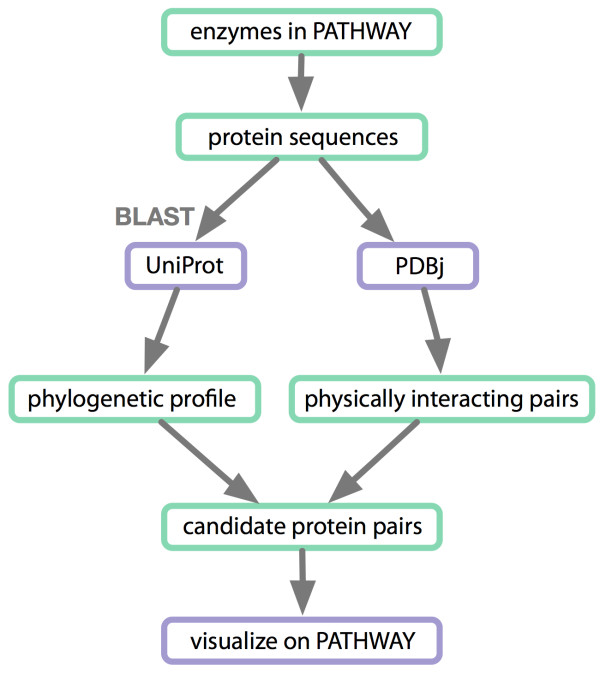
**Workflow to analyze protein interactions among enzymes in a KEGG pathway**. First, protein sequences are retrieved for each enzyme in a KEGG pathway. The sequences are then BLAST searched against UniProt and a phylogenetic profile is constructed of the results. Then, for each species in the phylogenetic profile, BLAST searches are run against PDB. Pairs of protein sequences (of the same species) that have homologs in the same PDB entry are inferred to be in physical contact and hence predicted to be interacting. Conserved and interacting proteins are then visualized on the pathway map, an example of which is shown in Figure 5.

**Figure 5:**
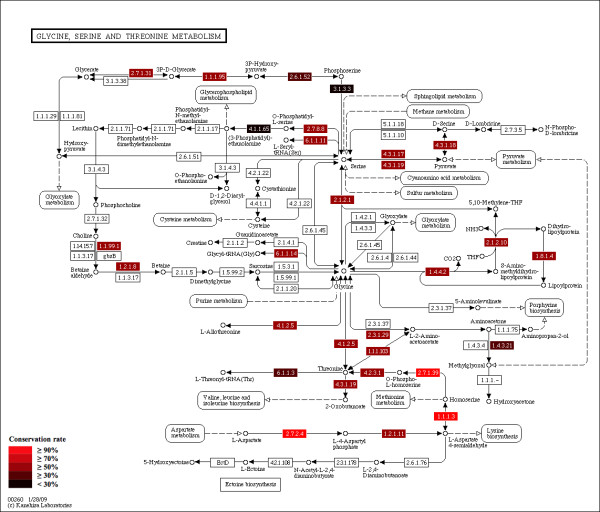
**Evolutionary conservation rate of proteins on a KEGG pathway**. Evolutionary conservation rate is defined as the ratio of the number of conserved proteins, i.e. homologs, over the number of species. Conservation rate is color-coded for each node in the pathway (see legend). See text and Figure 4 for more details.

A researcher wishes to enumerate putative physical protein interactions among enzymes in a KEGG metabolic pathway based on PDBj protein structure data. Here, participants constructed a workflow spanning DDBJ, KEGG, and PDBj which they implemented using a set of small script programs in Java, OCaml, Perl and Ruby. The workflow proceeds as follows: i) retrieve protein sequence of each enzyme in the user specified pathway from KEGG, ii) run a BLAST [45] search against UniProt [46] database for each sequence, iii) construct a phylogenetic profile, iv) run BLAST searches for each protein sequence against PDB for each species in the phylogenetic profile. If two protein sequences (of the same species) have homologs in the same PDB entry, they are inferred to be in physical contact, and hence predicted to be an interacting pair, v) output image files highlighting the conserved and interacting proteins in the pathway map. Figure 4 shows the steps in this workflow; an example of the produced visualizations is shown in Figure 5. To implement the workflow outlined above, participants first identified the SOAP and REST APIs of DDBJ [5], KEGG [10] and PDBj [8] for data retrieval. These were connected to each other and to the UniProt BLAST service using a workflow written mainly in Java.

### Use Case 4 - Analyzing glyco-gene-related diseases

**Figure 6:**
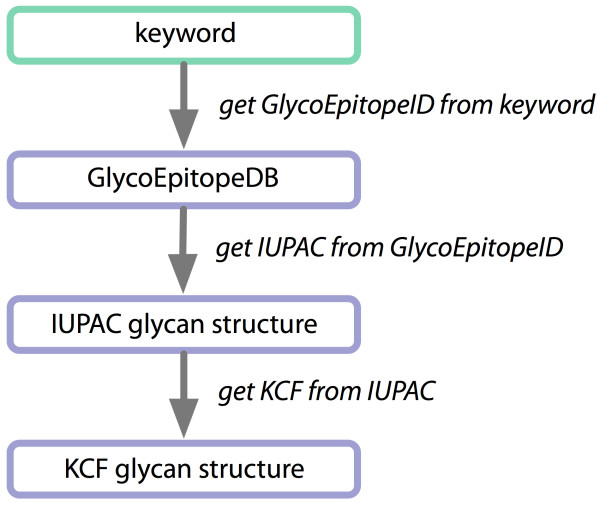
**Workflow for analyzing glyco-gene-related diseases**. In the first step of this workflow, GlycoEpitope DB entries are searched for disease-related keywords by a newly developed BioMoby service called *getGlycoEpitopeIDfromKeyword*. The identifiers of matching entries are then used to retrieve glycan structures in IUPAC format by another newly developed BioMoby service called *getIUPACfromGlycoEpitopeID*. The resulting IUPAC glycans are subsequently converted to KCF format by a new RINGS service called *getKCFfromIUPAC*. The KCF glycans can then be used for querying other RINGS data mining services.

A researcher needs to analyze glyco-gene-related diseases by searching for human homologs of glyco-genes in other species, such as fruit flies, and retrieving their phenotype-annotated SNPs. The approach was to use OMIM [47] to search for diseases related to loci of human homologs of target glyco-genes from other species, such as fruit fly, and retrieve any SNPs that are known to be related. To address this use case, participants agreed that the available Web services were insufficient, so as a starting point, participants first developed a workflow to retrieve H-Inv entries containing OMIM IDs for entries containing a particular disease-related keyword. Then, focus was on the development of Taverna workflows accessing existing and *de novo*-constructed Web services from the following glycobiology- related sites: GlycoEpitopeDB [48], RINGS [49], Consortium for Functional Glycomics [50], GlycomeDB [51], and GlycoGene DataBase [52], and an annotated database of human genes H-InvDB [53]. Two BioMoby [54,55] Web services for accessing GlycoEpitopeDB were constructed *de novo*: *getGlycoEpitopeIDfromKeyword *which queries GlycoEpitope DB using a keyword and retrieves all IDs of entries containing the keyword, and *getIUPACfromGlycoEpitopeID *which retrieves glycan structures in IUPAC format using GlycoEpitope IDs. In order to handle the data returned from these services, an additional Web service called *getKCFfromIUPAC *was developed in RINGS to convert a glycan structure in IUPAC format into KCF format, by which glycan structure queries could be made via other data mining Web services in RINGS. The steps of this workflow are visualized in Figure 6.

## Results

### Web services

The BioHackathon attendees identified Web services as a key technology for exposing valuable resources such as biological databases, algorithms or complex systems, to the whole research community. The nature of Web services allows them to be easily integrated via workflow tools and mashup applications. The problem of a required resource not being available as a Web service at all is a difficult problem, but it was addressed at the BioHackathon by the activities of the Daggoo [56,57] project in collaboration with the BioMoby project. Daggoo is a novel piece of technology that utilizes the strict data-typing environment of BioMoby as a way of leveraging semantic information from the behavior of biologists interacting with a Web page. In the Daggoo environment, users open the Web page that contains the analysis they wish to execute, and paste their data from the Daggoo clipboard into that Web page. Daggoo uses a set of regular expressions and rules, together with the BioMoby data-type ontology, to determine exactly what kind of data was pasted into which field in the Web form. It uses similar rules to automatically guess what kinds of data were produced by the invocation of that form. Subsequently, Daggoo is able to automatically create a BioMoby Web service wrapper around that Web form, and register that new service in the Moby Central registry. Once wrapped, the service becomes available for inclusion in a workflow like any other service. Thus, if the resource an end-user requires is not available as a Web service, it is possible for the end-users themselves to create this service simply by using the traditional Web interface of the resource provider. An extension of Daggoo that allows it to semi-automatically generate SADI-compliant Semantic Web services is near completion. With these tools available, the barrier encountered during our use-case analyses resulting from not having a tool or resource available as a Web service would be somewhat alleviated.

Also, among the Web service domain experts at this BioHackathon, developers of the G-language project implemented Web service interfaces to the G-language GAE in order to increase interoperability. The RESTful Web service [58] provides URL-based access to all functions of the G-language GAE, which makes them easily accessible from other tools. The SOAP interface provides a WSDL file [59] containing descriptions for all available programs in the G-language GAE. This WSDL can be readily loaded in Taverna 2 workbench, thus making these G-language functions available within Taverna workflows.

### Workflows

Researchers and developers of workflow tools used the BioHackathon to jointly investigate interoperability of their interfaces and evaluate their utilities by applying them to several real-world problems. Using data provided by the genomics researchers in attendance, participants addressed the powers, limitations and interoperability of a number of tools, including BioMart-Galaxy, BioMart-GBrowse, BioMart-Cytoscape and Galaxy-GBrowse. Collaborating with Galaxy, GBrowse and Cytoscape developers, those workflow system and data visualization tools gained better integration with BioMart Web services. Notably, established two-way communications within BioMart-GBrowse and Galaxy-GBrowse enable users to import data from and send data back to the relevant database from GBrowse. Furthermore, participants extended Galaxy to allow it to consume data from the TogoWS developed and maintained at DBCLS, which enables Galaxy users to easily incorporate several bioinformatics resources that TogoWS provides such as KEGG, DDBJ, PDBj, NCBI and EBI. The group also introduced internationalization codes into Galaxy so that non-ASCII characters can be displayed correctly and the interface can be localized for non-English native users.

Alongside more established workflow environments discussed here, SADI found its first public exposure to the wider bioinformatics community. SADI is a lightweight, standards-compliant Semantic Web services framework that utilizes Resource Description Framework (RDF) for its invocation and response messages, and Web Ontology Language (OWL) for its interface description. Matchmaking between data-holder and potential service providers - effectively, service discovery - is achieved through Description Logic reasoning. The novelty of SADI is that services must create meaningful relationships between the input and output data; as such, when a series of SADI services are invoked, the result is a network of inter-related data points which can be explored with a variety of visualization tools. Discussions between SADI developers and other participants led to the conclusion that providers of Web services should not be expected to do logical reasoning over incoming data; thus, the SADI API was modified such that all logical reasoning is now done on the client-side. This decision dramatically simplified the task of SADI Service provision, and this will make the framework more attractive to data and service providers in the future. Since the majority of workflow tools primarily facilitate manual workflow construction, the BioHackathon provided an opportunity to present work on semantically supported automated workflow construction to other workflow tool authors, and share ideas around successes and failures. These ideas are detailed in the "*Motivating arguments for semantics*" portion of the Discussion.

### Text mining

Participants investigated existing text-mining systems, such as automatic ontology-based tagging tools, and interfaces that enabled manual annotation of biological "objects" within life science research papers. During the BioHackathon, we explored automatic annotation services that include BioCreative MetaServer Platform [60,61], Whatizit [62] and iHOP [63,64], and collaborative annotation environments that include WiredMarker [65], XConc Suite [66], Kazusa Annotation [30] and Bionotate [67]. In addition, participants discussed how such annotation tools could facilitate the generation of networks of papers, based on an enriched set of metadata generated by them.

Participants noted that integrating automatic annotation tools with collaborative ones would bring about benefits to life science researches. Several groups have developed those tools that can be used for it and each one has its own strength. For example, Wired Marker and XConc Suite provide efficient manual annotation environments while Whatizit can annotate plain text on-the-fly. If an automatic annotation tool can be used through a collaborative one, annotation tasks will be more efficient. In addition, an ontology-mediated common data format adopted by several tools to represent annotated documents enables integration of various annotation results created by multiple study groups, and thereby literature or knowledge can be searched in more semantic ways.

### Visualization

During the BioHackathon a variety of tools were demonstrated, each having some novel and some overlapping visualization features. Among these was GenomeProjector's Google Map API feature, which provides three distinct aspects: genomic context, pathway context and DNA walk. Two demonstrated tools provide contextual semantic annotation based on the user zooming in ("semantic zoom"): these where genoDive and GBrowse. Yet another demonstrated approach is to explore genomes using huge, wall-sized projections. Lastly, in a tool developed at the BioHackathon, the exclusive Musical Instrument Digital Interface (MIDI) was used as a controller to navigate multi-dimensional, comparative genome data.

The developers of the Cytoscape plugin for querying PSICQUIC services, a protocol that allows the retrieval of molecular interaction data remotely from different sources and began its development in the previous BioHackathon, also presented their development progress. A REST interface was added to the service that enables streaming the results of a query obviating the need to retrieve the results in batches. In addition, it facilitates remote access to molecular interaction data repositories by using scripting languages or Web browsers.

## Discussion

The need to create new services in order to answer typical biological use cases speaks to two general problems in the bioinformatics Web service community: (1) the insufficiency of current offerings by data and tool providers, and (2) the difficulty data and service providers face in predicting what services are needed by their end-users. By solving common bioinformatics use-cases, participants discovered that many straightforward, yet non-obvious operations were lacking. For example, given a set of gene names, return a phylogenetic profile, or, given a set of BLAST hits, group them according to other shared features such as annotations or motifs. Clearly, if a researcher is constructing a workflow, and the next required function is not available as a Web service or it is embedded inside a more complex Web service, then a dead-end is reached in the workflow construction. This was observed repeatedly during the BioHackathon.

To resolve the pathway and glycoinformatics use cases, participants found that several services needed to be written *de novo*, in part because the required services did not exist, and in part because there were incompatibilities between Java and Perl Web services due to their respective SOAP libraries. Importantly, it was noted that most of the workflow was dedicated to file-format conversions (referred to as "shims" in the workflow community [68]), which in some cases led to building the workflows at the code level, rather than in a workflow design tool. Some conversions may be automated by newly developed Web services, for example using TogoWS's URL API for converting between a number of commonly-used flat file formats or to translate any flat file to JSON or XML (and in the future to RDF). However, these are nevertheless a nuisance for biologist end-users, and it is suspected that even with the availability of "shim" Web services, a non-trivial amount of coding for format conversions is inevitable as new technologies (and therefore new data formats) appear increasingly rapidly. It was also noted that the non-standard output format of BLAST search in PDBj sequence navigator caused additional problems. Taken together, these nuisances provided a strong lesson to data providers that they should, where possible, stick rigorously to standard formats for which tools exist.

One might speculate that at least part of the granularity problem in bioinformatics stems from the fact that a lot of data retrieval and analysis involves passing large, complex, opaque, and highly specialized flat-files. The observation of the proliferation of "shims" in bioinformatics workflows provides additional evidence for this problem. It perhaps should not be surprising, therefore, that the level of granularity of Web services in bioinformatics is generally at a level similar to the level of granularity of the flat files being passed around; i.e. there is very little incentive for service providers to offer Web services at any higher level of granularity than the flat files they understand, despite these finer-grained operations being extremely useful in the context of novel workflows. While this does not account for all cases of "blockage", participants observed while trying to construct workflows for the use cases that it was certainly a root cause for some of them. The corollary to this observation is that highly granular Web services lead to extremely complex workflows - complexity at a level that may well be beyond the capabilities of end-users. The trade-off between granularity and complexity might be resolved, at least partially, by the inclusion of "semantics" in data-types and Web services, such that workflow synthesis is more intuitive.

Given the difficulty in anticipating what Web services might be needed for any given research question, one might therefore speculate whether it will ever be possible for non-programmer end-users to build their analyses entirely in a workflow client; however, we contend there are ways to mitigate this problem given adherence to some simple best practices the participants identified at the BioHackathon and which we will outline below.

### Proposed guidelines

From these observations, participants have derived several guidelines that they will adopt as they continue to develop their respective tools; it is hoped that these guidelines will be useful to other groups facing similar problems. In brief, the biological Web service guidelines propose standard specifications for methods of REST and SOAP to search for, retrieve and convert formats of database entries. They also propose the format of query strings and recommended data types. Finally, they require the preparation of sample codes and documents. The full descriptions of these guidelines are available at the BioHackathon website [69].

While participants do not propose these guidelines as a formal international standard, it is strongly encouraged that bioinformatics resource providers examine these suggestions and follow those that are relevant to their own resources in order to maximize their usability and interoperability. The activity to maintain and update these guidelines continues, both as individual providers and as the wider BioHackathon community, and participants are continuing to observe and evaluate new technologies such as the Semantic Web and Semantic Web services.

An important conclusion from these guidelines is that participants believe resource providers should be as comprehensive as possible in making all data manipulation and analysis resources available as Web services - attempt to anticipate all the ways that users might want to manipulate data records and try to make these functions available as individual Web services. Also, resource providers should attempt to be as fine-grained and modular as possible in the Web services they implement. If a Web service, for example, applies a data transformation on the incoming data prior to executing its analysis, then providers should consider publishing both the transformation and the analysis as separate services, as it is likely that both functions will be independently useful in workflows that the provider cannot anticipate.

One might argue, however, that by adopting this approach the situation becomes worse by increasing the complexity of workflows. Fortunately, modern workflow tools such as Taverna allow users to publish sets of connected Web services as individual, reusable workflow modules. Thus, from the perspective of consumers, they have the ability to see whatever level of granularity they need to see - either the entire sub-workflow as a single function, or the sub-workflow as a set of highly granular and re-usable modules that they can splice together as they see fit.

### Motivating arguments for semantics

The discussions around semantics and the role of semantics in supporting automated workflow synthesis were diffused throughout the BioHackathon event, and there was considerable disagreement among the participants regarding the degree to which XML Schema and Schema-based Web services (i.e. WSDL) posed barriers to interoperability and integration, and the feasibility of alternatives.

Though the compelling argument in favor of XML Schema is the availability of a wide range of compatible tools, such tooling is rapidly becoming available for RDF/OWL also, thus this advantage becomes less significant every day. Moreover, as an increasing number of bioinformatics data providers begin publishing their data natively in RDF (e.g. UniProt [70]), it will soon become necessary not only to map XML Schema to one another to achieve interoperability, but also to map RDF-Schema to XML Schema (e.g. using SAWSDL [71]) in order to utilize traditional Web services. Representatives from the SADI project pointed out that this growing trend, in itself, should be sufficient motivation to look at alternatives to standard WSDL-based Web services.

Finally, our reliance on XML Schema has had other unintended consequences that not only thwart interoperability, but are making the problem worse over time. In a recent keynote address, Charles Petrie observed that "there are no practical Web services!" [72]. What he meant is that problems of data formatting, lack of annotation, lack of semantics, and the resulting complexity of workflow construction have all led to the situation where Web services are seldom modeled as modular units that can be "cobbled together" in various ways to achieve a variety of ends; rather, they are more often large multi-functional units with very low granularity (and therefore low re-usability in novel workflows). While Petrie was describing primarily business-oriented Web services, the situation in bioinformatics is not entirely dissimilar, and it is precisely this lack of fine-grained services that was identified by the BioHackathon participants as lacking. The movement to RDF/OWL-based data representation will, we propose, naturally lead to finer-grained data models and APIs simply because there is no advantage whatsoever in using RDF/OWL to pass complex, opaque documents. This, in itself, should provide the incentive to break these documents into richer, more granular structures, and this in turn will lead to the creation of more fine-grained APIs that can operate over them.

Increasing the granularity of the workflow almost invariably increases the granularity of data-types (e.g. serving only a small fragment of a PDB record, rather than the entire PDB record). The problem of data-type granularity has long been of interest to the BioMoby project. The data-type ontology in BioMoby was specifically designed to offer a much higher level of granularity in data representation than the typical bioinformatics flat-file, and it is this enhanced granularity that has enabled the creation of tools like Daggoo that can semi-automatically wrap existing Web resources. In practice, however, few data providers utilize the higher granularity of the Moby data-type ontology, preferring to simply consume and produce the same opaque flat-file formats they did before, but with a BioMoby object wrapper. As such, there was a general feeling at the BioHackathon that BioMoby did not offer a useful solution to this particular problem.

The use of RDF/OWL provides very strong incentives for data and service providers to increase the granularity of their data formats because, by doing so, it is possible to embed machine-readable *meaning *into that data. This added layer of meaning can be used, for example, by visualization tools to enhance the interpretation of that data by automatically selecting an appropriate rendering or representation for a specific piece of data based on its semantic properties, or could be used by workflow construction tools to automate the conversion of one data format to another equivalent or related data format, thus simplifying the "shim" problem. The SADI initiative is already demonstrating the advantage of using RDF/OWL in Web services.

## Conclusions

**Figure 7:**
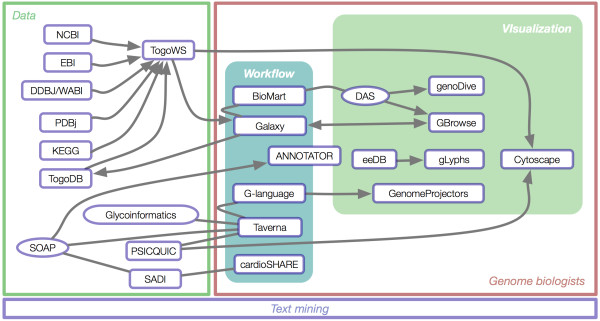
**Connectivity and compatibility of participating projects**. The BioHackathon 2009 was attended by participants representing projects operating in a number of problem domains (shown in Figure 1). Analysis of these participating projects during the hackathon revealed compatibilities and resulting connectivity as shown here.

The First DBCLS BioHackathon [2] focused on bringing data and analytical tool providers together with the goal of harmonizing their approach to making bioinformatics resources available as Web services (in the loosest sense of the term, including both programmatic APIs as well as REST-like services). The Second DBCLS BioHackathon brought together data providers, middleware providers, interface designers and end-users with the goal of evaluating our ability to address real-world biological problems through utilizing Web services to create data "mashups" with existing tools. In this evaluation, compatibility between many projects was leveraged, revealing a network of interoperable resources with high connectivity in some areas, as shown in Figure 7. However, the evaluation also revealed that there are still problems with the way we are modeling and annotating our Web services, including the lack of documentation of methods, lack of compliance with the SOAP/WSDL specification among and between various programming-language libraries, incompatibility between various bioinformatics data formats, and a dearth of useful data and analysis functions in Web services. Solving the real-world challenges posed by the biological researchers in attendance proved difficult to complete given these problems. Therefore, the need for semantic technologies was a common thread throughout the Second DBCLS BioHackathon, and it was determined that Linked Data and the Semantic Web initiative should be the core theme for the Third DBCLS BioHackathon.

## Competing interests

The authors declare that they have no competing interests.

## Authors' contributions

All authors attended the BioHackathon 2009. TK, MDW and RV primarily wrote the manuscript. TK, TaK, ShuK, MN, YY, HWC, AY, ShiK and TT organized the BioHackathon 2009. All authors read and approved the final manuscript.
